# *In Vivo* and *In Vitro* Study of a Polylactide-Fiber-Reinforced **β**-Tricalcium Phosphate Composite Cage in an Ovine Anterior Cervical Intercorporal Fusion Model

**DOI:** 10.1155/2011/109638

**Published:** 2011-10-26

**Authors:** Janek Frantzén, Aliisa Pälli, Esa Kotilainen, Harri Heino, Bettina Mannerström, Heini Huhtala, Hannu Kuokkanen, George K. Sándor, Kari Leino, Matias Röyttä, Riitta Parkkola, Riitta Suuronen, Susanna Miettinen, Hannu T. Aro, Suvi Haimi

**Affiliations:** ^1^Neurosurgical Unit, Department of Surgery, Turku University Central Hospital, P.O. Box 52, 20521 Turku, Finland; ^2^Institute of Biomedical Technology, University of Tampere, 33014 Tampere, Finland; ^3^Science Center, Tampere University Hospital, P.O. Box 2000, 33521 Tampere, Finland; ^4^Bioretec Ltd., Hermiankatu 22, 33720 Tampere, Finland; ^5^Tampere School of Public Health, University of Tampere, 33014 Tampere, Finland; ^6^Department of Plastic Surgery, Tampere University Hospital, P.O. Box 2000, 33521 Tampere, Finland; ^7^Department of Oral and Maxillofacial Surgery, University of Oulu, P.O. Box 5000, 90014 Oulu, Finland; ^8^Department of Anaesthesiology, Intensive Care, Emergency Care and Pain Medicine, Turku University Central Hospital, P.O. Box 52, 20521 Turku, Finland; ^9^Department of Pathology, Turku University Central Hospital, P.O. Box 52, 20521 Turku, Finland; ^10^Department of Radiology, Turku University Central Hospital, P.O. Box 52, 20521 Turku, Finland; ^11^Department of Eye, Ear, and Oral Diseases, Tampere University Hospital, P.O. Box 2000, 33521 Tampere, Finland; ^12^Department of Biomedical Engineering, Tampere University of Technology, P.O. Box 692, 33101 Tampere, Finland; ^13^Orthopedic Research Unit, Department of Orthopedic Surgery and Traumatology, University of Turku, Lemminkäisenkatu 2, 20520 Turku, Finland

## Abstract

A poly-70L/30DL-lactide (PLA70)–**β**-tricalcium phosphate (**β**-TCP) composite implant reinforced by continuous PLA-96L/4D-lactide (PLA96) fibers was designed for *in vivo* spinal fusion. The pilot study was performed with four sheep, using titanium cage implants as controls. The composite implants failed to direct bone growth as desired, whereas the bone contact and the proper integration were evident with controls 6 months after implantation. Therefore, the PLA70/**β**-TCP composite matrix material was further analyzed in the *in vitro* experiment by human and ovine adipose stem cells (hASCs and oASCs). The composites proved to be biocompatible as confirmed by live/dead assay. The proliferation rate of oASCs was higher than that of hASCs at all times during the 28 d culture period. Furthermore, the composites had only a minor osteogenic effect on oASCs, whereas the hASC osteogenesis on PLA70/**β**-TCP composites was evident. In conclusion, the composite implant material can be applied with hASCs for tissue engineering but not be evaluated *in vivo* with sheep.

## 1. Introduction

Degenerative or traumatic conditions of the cervical spine are generally treated by anterior cervical discectomy and fusion [1]. The highest fusion rates have been reported with this technique when the gold standard, autograft bone, has been used alone to replace the intervertebral disc [2, 3]. Nevertheless, this approach has major drawbacks, thus various biomaterials such as metals [3–5], polymers [6–8], and composites [9–12] for interbody fusion devices (IBFDs) are intensively studied. Metallic devices are a conventional option but not an ideal one due to several disadvantages such as cage migration, subsidence, nonunions, stress shielding, and artifacts in imaging methods [11]. Instead, polymer-ceramic composites are often utilized since the mechanical properties can be optimized according to a specific application [9, 13]. The polymer component provides strength, formability, and biodegradability, whereas the ceramic component provides bioactivity for the composite [13, 14]. 

The biodegradable polymers from the poly(*α*-hydroxy acid) family especially have been extensively investigated for spinal applications due to their excellent biocompatibility [15]. One of the most popular polylactides, poly-70L/30DL-lactide (PLA70), has been widely used for various applications, such as guided bone regeneration [16] and to repair large bone defects of the orbital wall [17] in addition to spinal defects. Furthermore, it has been applied successfully for bone fragment fixation, as well as for femoral and radial head fractures [13, 18, 19]. Especially the capability of PLA70 to tolerate sterilization without significantly impairing its mechanical properties [13] enables its versatile use in orthopedic applications. Importantly, the degradation products of PLA70 pins have been demonstrated *in vitro* and *in vivo* not to lead to any clinically relevant inflammation of either joint or lymph nodes [19]. 

Among bioactive components, *β*-tricalcium phosphate (*β*-TCP) has been used for decades in various orthopedic applications due to its osteoconductive and biodegradable nature [20]. Although *β*-TCP has several favorable properties, it has poor mechanical properties and hence an elevated risk of fracture. It is therefore more suitable as a filler material than to be used alone in load-bearing applications of major skeletal defects [14]. 

Although several intercorporal polymer-ceramic composites have been studied in spinal fusions, the ideal bioabsorbable weight-bearing fusion device that would promote fusion without causing foreign body reactions or biomechanical failures is yet to be found. In our study, we wanted to improve a previously introduced PLA70/*β*-TCP composite fusion cage [21], enhance its potential ossification properties, and increase its mechanical stability by incorporating circumferential poly-96L/4D-lactide (PLA96) continuous fibers around the composite. The primary aim of this study was to evaluate the *in vivo* bone formation ability of the novel fusion cage in ovine spinal fusion model. To assess the osteogenic potential and ensure the biocompatibility of the PLA70/*β*-TCP matrix material, we performed *in vitro* assays using human and ovine adipose stem cells (hASCs and oASCs).

## 2. Materials and Methods

### 2.1. Composite Implant Manufacturing for *In Vivo* and *In Vitro* Studies

The *in vivo* composite implants consisted of 3 different bioabsorbable raw materials: PLA70 (Boehringer Ingelheim GmbH, Ingelheim am Rhein, Germany) was used as a matrix polymer of the cage body, PLA96 (PURAC Biochem, Gorinchem, The Netherlands) as a reinforcement fiber material, and *β*-tricalcium phosphate (*β*-TCP, Plasma Biotal Ltd., Derbyshire, UK) as an osteoconductive filler in the PLA70 matrix. 

**Figure 1:**
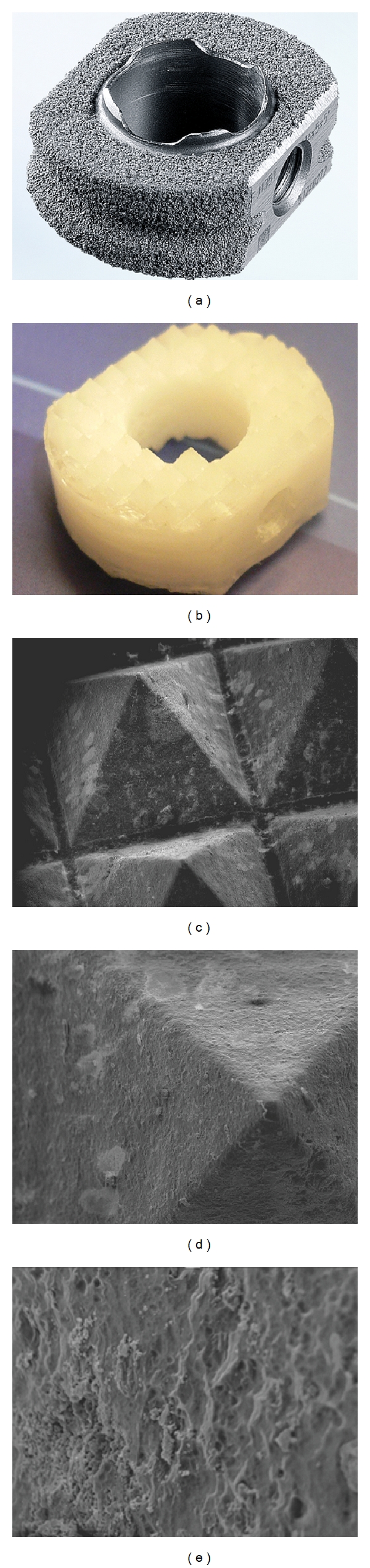
(a) CeSpace Titanium Plasmapore cage serving as a control implant for the *in vivo* study. (b) Poly-(70L/30DL)-lactide-fiber-reinforced *β*-tricalcium phosphate (*β*-TCP) composite cage (PLA70/*β*-TCP) for the *in vivo* study. (c), (d), and (e) Scanning electron micrograph (SEM) images of PLA70/*β*-TCP composite matrix by magnifications of 35X, 100X, and 500X, respectively, showing the ceramic particles in the surface of the polymer matrix without any isolating film.

The matrix polymer PLA70 and 30 wt% of *β*-TCP were first melt mixed using a 20 mm twin screw extruder. Preforms were machined out of the compounded matrix polymer rod containing 30 wt% of *β*-TCP. Reinforcing fibers made of PLA96 were melt processed using the same extrusion equipment and subsequently oriented to 0.3 mm thick fibers. Further, 2 meters of reinforcing fibers were wound around the preform. The hollow interior was machined after compression molding to the final implant shape including sharp ridges towards the vertebral endplates to ensure sufficient primary fixation. Due to this manufacturing technique, ceramic particles are not covered by the matrix polymer and are free to interact with surrounding fluids. (Figures 1(c), 1(d), and 1(e)). The size of the implant for the *in vivo* study was 14 mm × 16 mm with a lordotic 5° angle, and three different heights 5, 6, and 7 mm were used in order to accommodate different disc heights of the study animals. 

The* in vitro* composite samples consisted of 70 wt% of PLA70 and 30 wt% of *β*-TCP. The samples were manufactured similarly as the *in vivo* matrix composite implants. A custom made mold in the compression molding was used to ensure the uniformity of each sample. The polystyrene (PS) cell culture wells of 24-well plates (Nunclon Multidisk, Nunc, Roskilde, Denmark) were used as reference. The size of both composite samples and PS wells was equal (ø 15 mm).

Both *in vivo* composite implants and *in vitro *composite samples were manufactured by Bioretec Ltd. (Tampere, Finland) and gamma sterilized by Gamma-Service Produktbestrahlung GmbH (Radeberg, Germany) using 17.5 kGy irradiation. 

### 2.2. Control Implants and Other Hardware Used for the *In Vivo* Study

CeSpace Titanium Plasmapore pure titanium coated cages (Aesculap AG/Co. KG Tuttlingen, Germany) sized 14 mm × 11.5 mm with a lordotic 5° angle were used as control implants. The height of the implant was chosen according to the disc height ranging from 5 to 7 mm in 1 mm increments. For the primary stability through cranial and caudal fixation crown one-third tubular plate 3.5 w/collar, 5 holes, length 64 mm (SYNTHES Oberdorf, Switzerland), and cortex screws 3.5 mm diameter of 16–20 mm length (SYNTHES) were used in both study groups. One plate and two screws were applied for each fixation.

### 2.3. *In Vivo* Experiment in an Ovine Anterior Spinal Fusion Model

#### 2.3.1. Implantation Procedure and Followup

Ethical consent for the study protocol was given by the Animal Ethical Committee of the Provincial State Office of Western Finland (permit no. 1666/06), and all procedures were carried out in accordance with the guidelines of the local Animal Welfare Committee. Four 3-4-year-old sheep obtained from a class MV3 breeder (Reipelt, Finland) weighing 49–78 kg were used in this pilot study. The pilot animals were randomized to receive either the control implant (*n* = 2) or the composite implant (*n* = 2). Intramuscular (i.m.) premedication with midazolam was used. Fentanyl, ketamine, and propofol were administered intravenously (i.v.) for induction anesthesia, and propofol infusion was used to maintain anesthesia. Local xylocaine was sprayed into the larynx just before intubation. Amoxicillin 1 g was given before surgery i.v. 

Under strict sterile surgical conditions, each sheep underwent surgery for a single-level discectomy and anterior fusion C3/4. For autograft harvesting, an incision of 8 cm was made over the sternum. After partial reflection of the skin and subcutaneous tissues, using a 5 mm Kerrison punch (Aesculap AG/Co. KG Tuttlingen, Germany), 0.5 cm^3^ of autogenous cortical and cancellous bone was removed. The sternal incision site was closed using resorbable subcutaneous sutures Safil 2.0 (Aesculap, Corporate Parkway Center Valley, PA, USA) and stainless steel skin staples Appose ULC stapler (Autosuture/ Tyco Healthcare, Hampshire UK). 

Ventral exposure and discectomy were then performed. The level of interest was verified under fluoroscopy. A midline ventral incision was made, and the paired sternohyoid and sternomastoid muscles were separated on their midline. The longus colli muscle was incised in the midline, and the intervertebral disc C3/C4 was exposed and a complete discectomy was performed. The endplates were removed down to bleeding bone, using a high-speed burr. The hollow interior of the implant was then packed with autogenous bone graft harvested from the sternum and implanted under fluoroscopic imaging. The anterior stabilization with a one-third tubular plate and cortex screws was then performed in order to prevent extrusion of the implants due to excessive extension movements. Primary stability after implantation was ensured through the cranial and caudal fixation of the implant and by the anterior plate and screw fixation. Secondary stability was achieved through bony ingrowth into the increased surface area resulting from the control implants' Plasmapore coating.

The longus colli muscles aponeurosis, fascia, and subcutis were then closed in layers and the wounds were covered with a topical antibiotic powder, Bacibact (Bacitracin 250IU and Neomycin 5 mg, Orion, Finland) and liquid band-aid (Hansaplast, Hamburg, Germany).

Postoperatively, the animals received 0.01 mg/kg of buprenorphine i.m. 4 times daily for the first 2 days, and for the next 2 days twice a day. The health of animals was monitored twice daily throughout the study. The sheep were allowed *ad libitum* activity for the remainder of the followup. The pilot animals were sacrificed 6 months after the surgery. Euthanasia was achieved by exsanguination in anesthesia, and the spine segments of interest were harvested and cleared of soft tissue.

#### 2.3.2. Digital Radiograph

Standard anterior-posterior (A-P) radiographs of the spine segments were taken after surgery and before sacrifice on the digital image plates (Fuji IP cassette, Fuji Photo Film Co. Ltd., Japan) using a standard stationary X-ray unit (Philips, Holland). All sheep underwent computed tomography (CT) of the dissected bone block of the cervical spine using a 64-detector row CT system (Somatom Sensation 64, Siemens Medical Imaging, Erlangen, Germany). CT technical parameters included 140 mAs, 120 kV and 3 reconstructed image sets: axial slices with 1 mm section thickness and 1 increment with sharp kernel, coronal slices with 0.4 mm section thickness and 0.2 increment with sharp kernel, and sagittal 0.4 mm section thickness and 0.2 increment with sharp kernel. The matrix was 512 × 512 in all slice sets.

#### 2.3.3. Histology

The C3/C4 motion segments were fixed for 2–4 weeks in 10% normal buffered formalin followed by dehydration in ascending concentrations of ethanol. Clearing was performed in xylene for 4 days, and samples were then embedded undecalcified in isobornylmethacrylate (Technovit 1200 VLC, Kulzer, Algol, Germany).

Specimens were cut in the sagittal plane into approximately 5 *μ*m thick sections with a Leica heavy duty sliding microtome (Leica, Wetzlar, Germany). The sections were then stained with hematoxylin and eosin (H&E) and with modified van Gieson stains. Histologic sections were imaged using an Olympus B51 virtual microscope with a U-CMAD3 camera attached (Olympus Optical Co. Ltd, Tokyo, Japan). The foreign body reaction was graded according to the score of Hoffmann [22].

### 2.4. *In Vitro* Experiments

#### 2.4.1. ASC Isolation, Culture, and Seeding

hASCs from 2 donors were isolated from adipose tissue obtained from a surgical procedure at the Department of Plastic Surgery, Tampere University Hospital. oASCs from 2 sheep were provided by the Faculty of Medicine, University of Tampere. The ASC isolation from the tissue samples was conducted in accordance with the Ethics Committee of Pirkanmaa Hospital District, Tampere, Finland.

The minced tissue samples were digested with collagenase Type I (1.5 mg/mL; Invitrogen, Paisley, UK) as previously described [23, 24]. Isolated ASCs were cultured and expanded in T-75 cm^2^ flasks in maintenance medium consisting of Dulbecco's Modified Eagle Medium/Ham's Nutrient Mixture F-12 (DMEM/F-12 1 : 1 1x; Invitrogen), 10% fetal bovine serum (FBS, Invitrogen), 1% L-glutamine (GlutaMAX I, Invitrogen), and 1% antibiotics/antimycotic (100 U/mL penicillin, 0.1 mg/mL streptomycin, and 0.25 mg/mL amphotericin B; Invitrogen) [25–27]. As soon as the cells had reached confluence, they were stored at −70°C and thawed for the experiments. The immunophenotype of each ASC line was verified using flow cytometry (FACSAria, BD Biosciences, Erembodegem, Belgium) [28].

Cells of passages 3 to 4 were used for all experiments. Each composite sample was pretreated with maintenance medium for 48 h at 37°C in 24-well plates (Nunclon Multidisk, Nunc). The samples were seeded with 25,000 cells in a final culture volume of 1 mL of maintenance medium.

#### 2.4.2. ASC Attachment and Viability

Cell attachment and viability were evaluated qualitatively using live/dead-viability assay (Molecular Probes, Eugene, Ore, USA) at 7, 14, and 28 d time points. CellTracker Green 5-chloromethylfluorescein diacetate (CMFDA); Molecular Probes and ethidium homodimer-1 (EthD-1; Molecular Probes) were utilized to dye viable cells (green fluorescence) and dead cells (red fluorescence), respectively [25–27].

#### 2.4.3. Cell Proliferation and Quantitative Analysis of Alkaline Phosphatase Activity

On the day of the analysis, 250 *μ*L of 0.1% Triton-X 100 buffer (Sigma Aldrich, St. Louis, MO, USA) was added to each well and thoroughly suspended. The well-plates were stored at −70°C at least over night to lyse the cells. The DNA content of ASC composite and PS samples was measured after 7, 14, and 28 d culture periods using a CyQUANT Cell proliferation assay kit (Molecular Probes—Invitrogen) according to manufacturer's protocol and as earlier described [29]. The fluorescence was measured with Victor 1420 Multilabel Counter, (Wallac, Turku, Finland). The quantitative alkaline phosphatase (ALP) measurement was performed at 14 and 28 d time points according to the Sigma ALP procedure (Sigma Aldrich) [26] with minor modifications. Quantitative ALP activity results were normalized to the total DNA amount measured from the same lysates.

#### 2.4.4. Mineralization

Von Kossa staining was used to qualitatively estimate the mineralization nodule formation to demonstrate the osteogenic differentiation of ASCs. A previously described method [30] was used with minor modifications. Briefly, the cultures were fixed with 4% paraformaldehyde for 2 min, followed by a double wash with distilled water. A 5% silver nitrate (AgNO_3_) solution was added to the wells, and the plates were incubated under an ultraviolet (UV) lamp for 60 min without covers. AgNO_3_ was removed, the cultures were washed with distilled water, and the plates were photographed with a digital camera.

#### 2.4.5. Statistical Analysis of the *In Vitro* Results

The statistical analyses were performed with SPSS, version 14. The data was presented as mean ± standard deviation (SD) for both quantitative analyses. Equal variance assumption was checked by Levene's test. All statistical analyses were performed at the significance level *P* < 0.05 using one-way analysis of variance (ANOVA) or *t*-test. Bonferroni post hoc correction for multiple corrections was used. The effects of different culturing times (7 versus 14 versus 28 d) and sample materials (composite versus PS) were evaluated for both species. Samples were regarded as independent at each time point.

## 3. Results

The postoperative healing was uneventful except for 1 animal from the composite implant group that suffered ORF virus disease. Due to weakness of the hind legs, the animal had to be euthanized 5 months after surgery.

### 3.1. Radiographic Evaluation of Fusion

**Figure 2:**
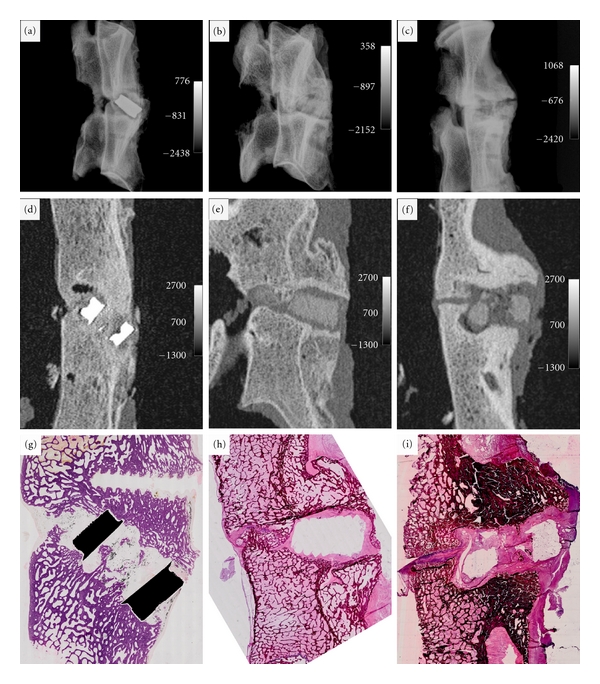
(a–c) Lateral plain X-rays showing aligned vertebrae at 6-month time point ((a) for control implant and (b)-(c) for composite implants). (d) Sagittal CT at the center of the control implant shows an advanced fusion. (e) Sagittal CT image shows no implant to bone contact or fusion of the adjacent vertebral bodies of the composite implant. (f) Sagittal CT image at the centre of the composite implant showed fragmentation of the implant but no migration to the spinal canal and malunion of the vertebras. (g) Hematoxylin and eosin (H&E) staining of a sagittal section at the centre of the control implant shows bone ingrowth through the implant and signs of dark fine granule surrounding the implant suggesting metallosis. (h)-(i) A dense fibrotic capsule and a mild foreign body reaction surrounded the composite implants, and no bone contact was found.

Lateral plain X-rays showed well-aligned vertebrae in all study groups (Figures 2(a)–2(c)). CT images of the composite implant group showed fragmentation in one implant and one anterior coronal fracture line in the other implant, but no migration of the fragments. No continuous bone growth through the implant was observed, and no implant to bone fusion was found (Figures 2(e)-2(f)). There was a marked resorption of the autograft. Sclerosis of the endplates was observed suggesting malunion. No signs of osteitis were seen in any of the study groups. In the control implant group an advanced fusion was seen inside the implant, with trabecular orientation and almost complete integration of the autograft to the adjacent vertebra (Figure 2(d)).

### 3.2. Histomorphological Studies

Histomorphological analysis of both groups at 6 months supported the findings of the radiographic examinations. Histologically, a mild foreign body reaction developed in the composite implant group, according to the score of Hoffmann [31]. There was a marked resorption of the autograft and multinucleated foreign body macrophages inside the implant. A dense fibrotic capsule surrounded the composite implant, and no bone contact was found (Figures 2(h)–2(i)). Furthermore, no signs of acute infection were found. One of the implants was fragmented at 6 months followup, but no migration of fragments was observed. The endplates were dense, and the expected trabecular orientation at the fusion site had disappeared suggesting malunion. 

After 6 months, there was an advanced interbody fusion in the control group seen as bone ingrowth through the implant. Improved alignment in the vertical and horizontal trabeculae was observed. There was a direct bone contact at the bony endplates to the implant. There were signs of dark fine granules surrounding the implant, which were visible in Polaroid light suggesting metallosis (Figure 2(g)).

### 3.3. *In Vitro* Experiments

#### 3.3.1. ASC Characterization

The flow cytometric analysis on ASCs demonstrated expression for markers substantiating mesenchymal origin of cells and showing low or lack of expression of markers suggesting hematopoietic and angiogenic origin of cells (Table 1). The characterization data comply with existing results on hASCs [23, 28, 32–35] and oASCs [36–38].

#### 3.3.2. Cell Viability

**Figure 3:**
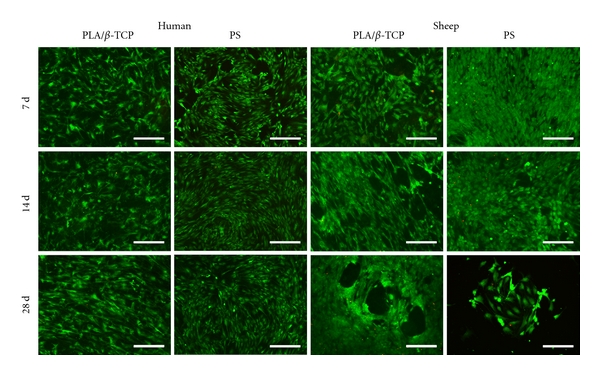
Representative images of viable (green fluorescence) and dead (red fluorescence) adipose stem cells (ASCs) attached to composite and polystyrene (PS) samples. Scale 100 *μ*m.

Microscopic observations revealed that after 7 days of culture, the ASCs of both species had normal morphology and they were well attached to the composite samples and PS (Figure 3). The majority of the cells of both species were viable, and the number of dead cells was low. hASCs were homogenously spread on both composite samples and PS. By contrast, oASCs tended to detach more from the composite surfaces at all time points than hASCs. In addition, the number of oASCs was estimated to be higher on PS than composite samples. The high proliferation rate of oASCs and the limited growing space eventually led to complete detachment of oASCs at 28 d in some PS wells as shown in Figure 3.

#### 3.3.3. Cell Proliferation and Osteogenic Differentiation

**Figure 4:**
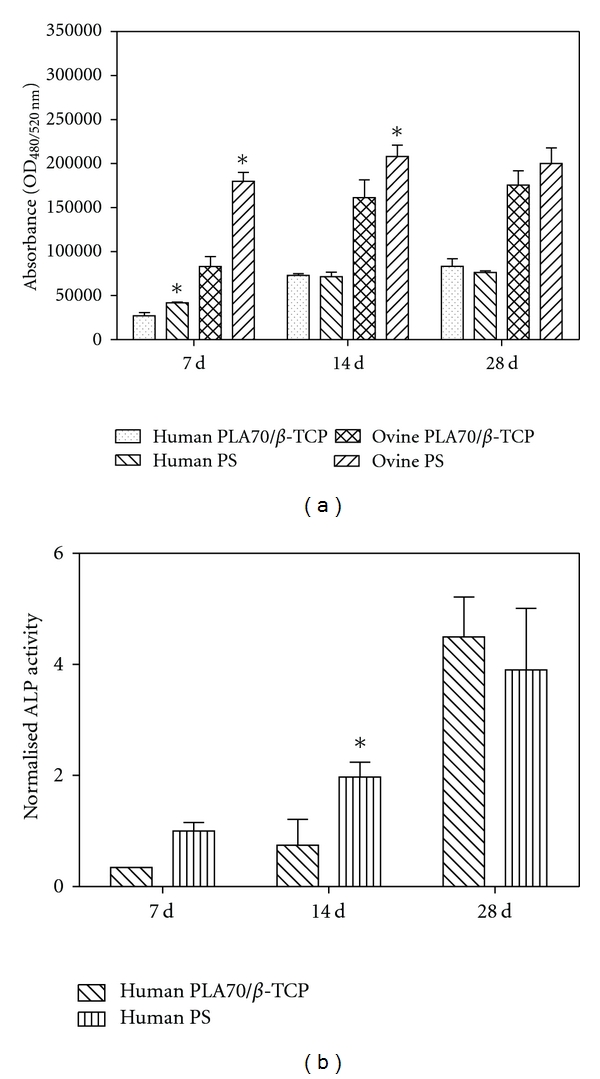
(a) Relative DNA content of adipose stem cells cultured for 7, 14, and 28 d on different materials measured with CyQUANT proliferation assay kit. The optical density (OD) was measured at 480/520 nm. Results are expressed as mean ± standard deviation (SD), *n* = 4. *P < 0.05 with respect to corresponding composite implant (PLA70/*β*-TCP). (b) Alkaline phosphatase (ALP) activity of human adipose stem cells cultured for 7, 14, and 28 d on different materials measured with ALP activity kit. Results are expressed as mean ± SD, *n* = 4. *P < 0.05 with respect to corresponding PLA70/*β*-TCP.

The number of ASCs present on both samples was assessed quantitatively using the CyQUANT Proliferation method (Figure 4(a)), which is based on the relative absorbance values of the amount of DNA. The number of oASCs was higher at each time point compared to the hASCs on both materials. In addition, the number of hASCs was significantly higher on PS compared to the composite samples only at the 7 d time point (*P* < 0.001), whereas the number of the oASCs was higher on the PS during the whole culture period. This difference was significant at the 7 (*P* < 0.01) and 14 d time points (*P* = 0.008). Further, the number of hASCs had increased significantly only between the 7 and 14 d time points on both materials (*P* < 0.01). The same phenomenon was seen with oASCs, but only on composite samples (*P* < 0.01).

According to the ALP activity analysis, oASCs did not show any signs of osteogenic differentiation. The normalized ALP activity of hASCs increased over time on PS (Figure 4(b)). On the composite, the increase in ALP activity was significant between the time points of 14 and 28 d (*P* = 0.004). The relative ALP activity of hASCs was higher on composite at 28 day, than the PS, but the difference was not statistically significant. 

**Figure 5:**
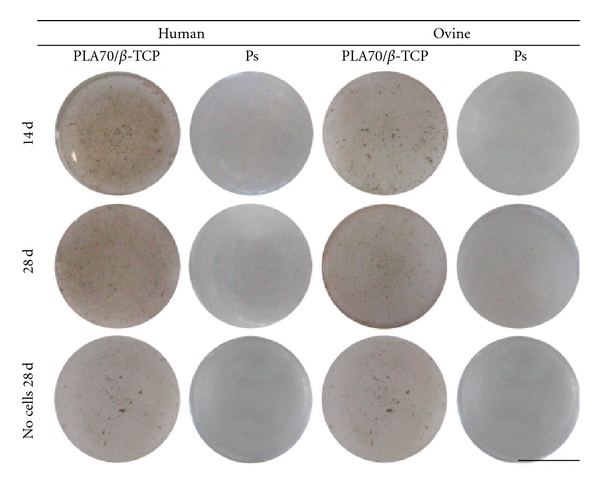
Von Kossa staining of human and ovine adipose stem cells at 14 and 28 d time point. Scale 75 mm.

The von Kossa staining demonstrated that the PS had no effect on the mineralization of ASCs from either of the species at any time point (Figure 5). The hASCs mineralized on the composites, verified by the dark staining of granules. However, there was no difference between the time points. The staining of hASCs was more intensive compared to oASCs that only demonstrated a minor staining result. 

## 4. Discussion

This study evaluated the *in vivo* bone formation efficacy of a PLA96-fiber-reinforced PLA70/*β*-TCP composite cage in a head-to-head comparison with a titanium cage that is widely used in clinical practice. The *in vivo* model chosen for this project was the ovine cervical fusion and discectomy model due to its wide use in spinal studies [8, 31, 39, 40]. Sheep continue to be among the most popular animal models for orthopaedic research due to their similarities to human, especially weight, size, joint structure, and bone/cartilage regenerative process [41]. 

Clinical evidence exists showing that different PLA/*β*-TCP composites are biologically safe and that they may be feasible alternatives as materials for bioabsorbable spinal fusion implants [10, 42–44]. Accordingly, the composite implant used in this study has very similar modulus of elasticity to that of the trabecular bone and is therefore a beneficial choice with regard to stress shielding. Along with material resorption, additional load is transferred to the developing mass. 

Studies have shown higher fusion grades using osteoinductive agents, such as bone morphogenetic protein 2 (BMP-2) instead of autograft bone [7]. In this study, we attempted to investigate material properties and chose to use autograft filler in both cohorts. Moreover, anterior plating is mandatory in the ovine model, thus making the attempted fusion site even more rigid. As several clinical studies suggest, this leads to increased fusion and reduced failure rates, particularly in multilevel procedures [45, 46]. Nevertheless, the present study yielded poor results in ossification of the composite implant in an ovine fusion model, whereas the control implant yielded a remarkably better ossification of the vertebrae. This result was clear, although we acknowledge that the study was limited by the small number of animals and time points. 

One of the possible causes for the failure was the premature degradation of the composite implant as indicated by the malunion detected in the radiographic examination. For the same reason, the composite implant did not provide sufficient structural support to allow fusion of the treated segment. The poor osseous integration of the composite implant itself would predispose to pseudoarthrosis by allowing inordinate movement in the segment. The single anterior plating used in this study would not provide sufficient rigidity to fuse the segment; instead, the primary task was to avoid extrusion of the implant by eliminating the excessive extension of the treated segment. A mild foreign body reaction was observed in the composite implant group, but it was not considered a reason for the malunion. Even though a dense fibrotic capsule had developed around the composite implant, it had formed secondarily to the malunion. In addition, macrophages were detected inside the implant, which was conceived as a sign of autograft resorption in the inner site of the composite. As a comparison, Kandziora et al. [11] studied the composite fusion cage of polymer-calcium phosphate, which showed even higher level of foreign body reaction by the Hoffman grade than in this study and superior biomechanical properties compared to tricortical bone, though not interfering the formation of bony union in a sheep cervical spine fusion model. These results further suggest that the foreign body reaction cannot explain the malunion in this study. The degradation time of the composite implant could further be increased to provide a longer safety margin if osseous union formation is retarded. It is, however, clear that osseous union is of the utmost importance for this type of fixation and longer degradation time most likely does not yield better outcome, just failure at a later phase. In order to facilitate fusion and provide sufficient structural support, slightly slower degrading polymers, such as PLA96 as matrix and poly-L-lactide (PLLA) fibers, could be chosen for longer strength retention time. In the ovine model, one has to take into account that the ovine body temperature is three degrees higher than that of humans resulting in a remarkably higher degradation than suggested by calculations based on the Arrhenius equation. With respect to the composite material, both *in vivo* and *in vitro* composites were machined and subsequently compression molded in precisely the same way leading to uniform surface structures without any isolating polymer film, which would have hindered the bioactive *β*-TCP component from being at the surface.

To further ascertain why the ossification of the composite implants was unsuccessful, *in vitro* biocompatibility and osteoconductivity assays with ASCs from both human and sheep were performed. We used ASCs over osteoblasts to evaluate the capability of *β*-TCP in inducing osteogenic differentiation of these mesenchymal stem cells. The osteogenic potential of hASCs is well characterized from earlier studies [47–49], but it has not been extensively studied with oASCs. However, the osteogenic differentiation potential of ovine-derived adult stem cells has been verified: Rhodes et al. with ovine bone-marrow-derived mesenchymal stem cells (bMSCs) when cultured in 3D hyaluronic scaffolds [50] and Niemeyer et al. with oASCs when differentiated *in vitro* without any biomaterial [51]. In both studies, the cells were treated with osteogenic differentiation factors during cell culture. It should be noted that no osteogenic medium was used in our study, thus only the osteogenic effect of the composite material was evaluated. 

 In the *in vitro* part, we chose to focus on PLA70/*β*-TCP matrix as it was considered the most important component of the composite implant with regard to bone formation. PS was used for *in vitro* assessment control to better evaluate standard cellular behavior. The *in vitro *studies showed that there was no difference between hASC and oASC viability on the composite samples and PS as indicated by the small number of dead cells and normal attachment on the surfaces. Conversely, the proliferation rate of oASCs was notably higher than that of hASCs, which partly led to the detachment of oASCs due to the lack of growing space. On the other hand, the number of oASCs was greater on PS at each time point, whereas the hASCs grew equally on both PS and composite. The osteogenic differentiation of hASCs was verified on composite samples, as detected by the ALP activity measurement and the von Kossa staining. This result was consistent with our earlier study, where we showed that the PLA/*β*-TCP composite scaffolds enhanced the osteogenic differentiation of hASCs over the plain PLA scaffolds [27]. Interestingly, the von Kossa staining of oASCs demonstrated a weak mineralization result, whereas the PLA70/*β*-TCP matrix failed to induce the osteogenic differentiation of the oASCs according to the ALP activity measurement. Our results suggest that the PLA70/*β*-TCP does not support the osteogenic differentiation of ovine mesenchymal stem cells derived from adipose tissue. Consequently, it may partly explain the unsuccessful osseointegration results obtained in the *in vivo* study. This conclusion, however, should be further verified with other mesenchymal stem cells. 

In a clinical perspective of bone tissue engineering applications, autologous cells are the best choice, also for products that involve scaffolding. It is important to note that if similarities or differences between ASCs from different species remain unknown, the information about the cellular characteristics cannot be exploited for an ideal preclinical study setup. Given such knowledge, it is possible to choose a suitable animal model for each bone defect application treated by tissue engineered constructs and subsequently evaluate the usability of autologous ASCs.

## 5. Conclusions

This study failed to demonstrate the *in vivo* bone formation capability of the PLA70/*β*-TCP/PLA96 composite fusion cage in the chosen ovine model. According to the *in vitro* results, the PLA70/*β*-TCP composite matrix material induced only a weak osteogenic response on oASCs, whereas the osteogenic effect of the material on hASCs was strong.

## Figures and Tables

**Table 1:** Phenotypic characterization of human (hASC) and ovine (oASC) adipose stem cells by flow cytometric analysis of 10,000 cells. Data are presented as mean ± standard deviation (SD).

Surface protein	Manufacturer	hASC expression (mean ± SD)	oASC expression (mean ± SD)
CD14	BD	5.4	8.0 ± 2.4
CD19	BD	1.5	1.2 ± 0.1
CD34	IT	4.3 ± 0.9	23.6 ± 14.5
CD45	BD	1.1 ± 1.2	9.3 ± 4.2
CD105	RD	87.3 ± 11.4	30.3 ± 7.2
CD117	MB	Not analyzed	0.5 ± 0
HLA-DR	IT	0.5	1.8 ± 0.3

BD: BD Biosciences, Erembodegem, Belgium; IT: Immunotools GmbH Friesoythe, Germany; RD: R&D Systems Inc., Minn, USA; MB: Miltenyi Biotech, Bergisch Gladbach, Germany; Sigma: Sigma, St. Louis, Mo, USA; HLA-DR, major histocompatibility class II antigen.
